# Development of a Novel Guided Wave Generation System Using a Giant Magnetostrictive Actuator for Nondestructive Evaluation

**DOI:** 10.3390/s18030779

**Published:** 2018-03-04

**Authors:** Mingzhang Luo, Weijie Li, Junming Wang, Ning Wang, Xuemin Chen, Gangbing Song

**Affiliations:** 1 School of Electronics and Information, Yangtze University, Jingzhou 434023, China; lmz@yangtzeu.edu.cn (M.L.); cjdxwjm@yangtzeu.edu.cn (J.W.); 2 Department of Mechanical Engineering, University of Houston, Houston, TX 77204, USA; wli27@uh.edu; 3 Department of Engineering, Texas Southern University, Houston, TX 77004, USA; chenxm@tsu.edu

**Keywords:** giant magnetostrictive (GMS) actuator, ultrasonic guided waves, rock bolt monitoring, nondestructive evaluation

## Abstract

As a common approach to nondestructive testing and evaluation, guided wave-based methods have attracted much attention because of their wide detection range and high detection efficiency. It is highly desirable to develop a portable guided wave testing system with high actuating energy and variable frequency. In this paper, a novel giant magnetostrictive actuator with high actuation power is designed and implemented, based on the giant magnetostrictive (GMS) effect. The novel GMS actuator design involves a conical energy-focusing head that can focus the amplified mechanical energy generated by the GMS actuator. This design enables the generation of stress waves with high energy, and the focusing of the generated stress waves on the test object. The guided wave generation system enables two kinds of output modes: the coded pulse signal and the sweep signal. The functionality and the advantages of the developed system are validated through laboratory testing in the quality assessment of rock bolt-reinforced structures. In addition, the developed GMS actuator and the supporting system are successfully implemented and applied in field tests. The device can also be used in other nondestructive testing and evaluation applications that require high-power stress wave generation.

## 1. Introduction

A guided wave can propagate in many different waveguide structures, such as plates, rods, pipes, and multilayer structures [1,2]. Because of the effects of reflection, refraction, and mode conversion on the waveguide boundaries, constructive and destructive interferences occur during the propagation of the waves [3,4]. The waves are then superposed and the guided waves are formed in the waveguide structure. The field of ultrasonic guided waves has created much interest in the past decades. The number of publications, research works, activities, and actual applications has increased significantly [5,6,7,8,9,10,11]. The propagation characteristics of the guided waves are often closely related to the structure, the defect type, and the mechanical properties of the material [5,12]. Therefore, there are many application areas for guided waves, such as seismology [13], inspection [14], biomedical engineering [15], concrete [16], material characterization [17], and even in the area of communication [18].

Ultrasonic guided waves in solid media become a critically important subject in nondestructive evaluation (NDE) and structural health monitoring [19,20], and in many other engineering fields [21,22]. Among them, damage localization based on the propagation of ultrasonic guided elastic waves is one of the most typical applications in various structural elements [19,23]. New, faster, more sensitive, and more economic ways of looking at materials and structures have become possible when compared to the previously used normal beam ultrasonic or other inspection techniques. With these techniques, for example, the process of inspecting an insulated pipe required removing all the insulation, and using a single probe with a normal beam to check along the length of the pipe with thousands of waveforms. Now, one can use a guided wave probe at a single location, leaving the insulation intact, to inspect the segment of a pipeline by examining just a few waveforms [24]. All this has become possible through the development of guided wave theory and technology. The spectral finite element method has been proposed as an efficient simulation technique, which provides an analytical framework to predict guided wave propagation [25,26], and many researchers focus on the application of guided wave methods and the spectral finite element in structural monitoring [27,28]. Taking the environmental factors into account during the application of guided wave in structural health monitoring, some integration and fractal signal processing methods are proposed [29,30]. Rock bolts, often with a length of several meters, are the major supporting elements for underground structures, such as tunnels, dam foundations, and underground excavations. There are more than 500 million rock bolts used worldwide every year [31]. Rock bolt failures are the major causes for collapses of these structures. Therefore, it is highly desirable to develop a portable stress wave generation device with a high action energy, to enable stress wave-based nondestructive evaluation of rock bolts.

As a new type of functional material, giant magnetostrictive material has many advantages, such as high magnetostriction coefficient, high energy density, low magnetic field drive, high magnetic conversion efficiency, and fast response [32]. Intensive research on giant magnetostrictive (GMS) materials has led to broad applications in several potential fields [33,34,35]. Giant magnetostrictive materials have strong magnetostrictive effects and magnetostrictive reactions, and show two-way energy conversion characteristics during operation. When the ferromagnetic material is subject to an external magnetic field, the change of magnetization state causes small changes in the size, resulting in a magnetostriction effect [36]. The actuator can be fabricated into different dimensions, realizing force, microdisplacement drive, or vibration control. This kind of actuator has the following advantages: wide temperature range, low voltage operability, no cable drive, large output force, relatively smooth frequency response, fast response speed, and high control precision [37,38]. According to its output form, it can be classified as a microdisplacement actuator and a force actuator, such as a GMS microdisplacement actuator and a micromagnetostrictive vibrator, among others [39,40]. It is possible to use GMS material as an actuator for guided wave generation [41,42,43]. However, those reported in the literature are normally for special purposes, and often require installation. For many applications, such as the measurement of the length of embedded rock bolts and road guards, there will be a large number of involved specimens, which requires the inspection to be quick and convenient. Often, it calls for inspection upon contact without using any coupling agent. Some specimens are very long, often more than 3–5 m, which demands stress waves with high power. In general, the stress wave-generating unit should be powerful, portable, and convenient to use (i.e., measurement upon contact), and should require no coupling agent.

In this paper, a new type of guided wave actuator device was developed specifically for stress wave generation with a large output force, based on the giant magnetostrictive (GMS) effect. A key innovation of the proposed new GMS actuator design involves a conical energy-focusing head that can focus the amplified mechanical energy generated by the GMS actuator. This design enables the generation of stress waves with high energy, and the focusing of the generated stress wave on the specimen to be tested. In addition, the design enables measurement upon contact without the use of a coupling agent between the actuator and the testing specimen. The supporting electrical system was implemented by using pulse code modulation (PCM) and direct digital synthesis (DDS) driving technology. The developed device supports two kinds of output modes: pulse coding and scanning signal. The developed system has the advantages of a large driving force, adjustable output power, wide bandwidth, low requirements upon coupling, simple operation, and being portable. The effectiveness of the developed system was demonstrated in the quality assessment of rock bolts through stress wave-based nondestructive evaluation, in both laboratory and field settings. The device can also be used in other NDE applications that require stress wave generation.

## 2. A Review of Methods for Guided Wave Generation

In the use of ultrasonic guided waves for nondestructive evaluation, we need to study the effects of different excitation signals and signal processing methods on the detection results [44,45,46]. Coded excitation is an effective means to ensure a high signal-to-noise ratio, and thus improve detection accuracy [47]. There are three kinds of commonly used ultrasonic methods for guided wave generation; namely, piezoelectric, electromagnetic, and pulsed laser methods.

### 2.1. Piezoelectric Transducers

Guided wave excitation by the piezoelectric method is very common in practical applications [48,49]. A piezoelectric transducer works based on the effect of piezoelectricity to generate mechanical waves. When mechanical stress or strain is applied, the piezoelectric material will generate electrical charge. Conversely, when an electric field is applied, the piezoelectric material will produce mechanical stress. Commonly used piezoelectric materials include piezoelectric crystals, piezoelectric ceramics, and piezoelectric composite materials [50,51]. These materials can be made into a piezoelectric film or a complete package of piezoelectric probes. The mechanical vibrations generated by the piezoelectric material are transmitted to the waveguide structure through the coupling medium. Therefore, the coupling condition has a direct effect on the performance of the transducers.

### 2.2. Electromagnetic Transducers

The electromagnetic guided wave generation method is mainly used to inspect conductive materials and ferromagnetic materials. The mechanical vibration is generated through the Lorentz force or the magnetostrictive effect. The electromagnetic transducer usually consists of a coil, a magnet, and conductive or ferromagnetic components. Different wave types, such as Rayleigh waves, Lamb waves, and SH waves can be generated, based on the direction of the coil and the magnetic field [52,53]. The Lorentz force-based electromagnetic transducer is more suitable for the inspection of nonferromagnetic conductive materials, while the magnetostrictive-type electromagnetic transducer is more suitable for use with ferromagnetic materials. In fact, the excitation energy generated by the magnetostrictive effect is much greater than that generated by the Lorentz force effect. Therefore, researchers have designed the guided wave transducer by embedding the magnetostrictive material into the transducer [54,55].

### 2.3. Pulsed Lasers

Laser-based ultrasound is the use of a pulsed laser to impact a solid surface, where the ultrasound is generated through ablation and thermoelastic effects. Compared with the piezoelectric and electromagnetic types of ultrasonic transducers, this type of transducer has fewer excitation modes. It is limited to point source, line source, and arrayed line source excitation [56]. Laser-based ultrasound can be used to excite Lamb waves and Rayleigh waves in plate structures [57,58]. This method requires expensive high-energy laser devices.

### 2.4. Comparative Analysis

These three methods have their own advantages and disadvantages. The piezoelectric method has the advantages of a high energy conversion rate and low price; however, its performance is influenced by the coupling condition and by limited strain and energy density. The laser-based guided wave generation method is a noncontact method, which is favorably used for inspecting complex components. The advantage of this method is its high resolution, but it has the disadvantages of requiring expensive equipment and complex operation. It is not suitable for field applications. Alternatively, if resolution is not a primary concern, guided waves can be generated by a hammer impact. The hammer impact method requires highly-trained personnel. It is very difficult to ensure the consistency in excitation energy, frequency, and phase for each impact, which is unfavorable for postsignal processing. With the development of GMS materials in the past few decades, the magnetostrictive method has the advantages of a high extension rate, high energy density, rapid response, wide bandwidth, and the ability to excite many modes of guided waves. With proper design, there is no need for a coupling agent between the actuator and the testing structure. This method has the potential to replace the piezoelectric-based method in many application areas. There is a need to develop a GMS stress wave generation system specifically for NDE, which requires high actuation power.

## 3. The Design of the Novel GMS Actuator System

The proposed giant magnetostrictive ultrasonic guided wave transducer is made from a rare earth alloy (a Tb–Dy–Fe alloy). It has the advantages of a wide frequency range, small size, excellent repeatability, and most importantly, large actuation stroke and power, which together meet the requirements of guided wave generation for the NDE of rock bolts.

### 3.1. Principle Analysis and Mechanical Design

**Figure 1:**
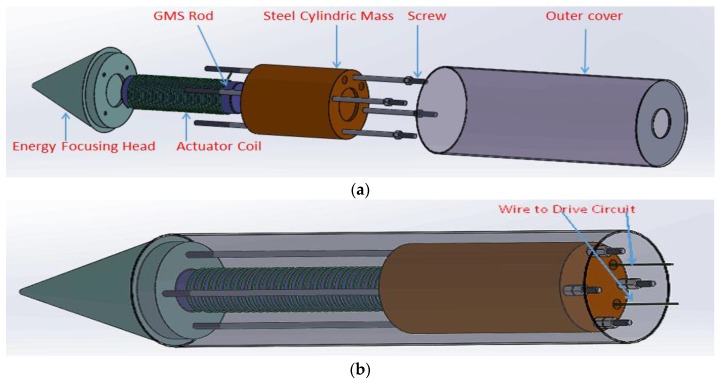
The giant magnetostrictive (GMS) actuator. (**a**) Components of the GMS actuator; (**b**) Assembled GMS actuator.

Conventional giant magnetostrictive actuators are designed to harvest large displacements. Since the purpose of the proposed actuator is for stress wave generation, a new mechanical design had to be developed. Figure 1 shows the components and the assembly of the GMS actuator. The proposed GMS actuator consists of a GMS rod, an actuator coil, an energy-focusing head, a cylindrical steel mass, and long connecting screws. The GMS rod is placed between the energy-focusing head and the steel mass. All the components are assembled using the long connecting screws. The GMS rod was coated with a protective silver coating before being placed in the center of the actuator coil. The supermagnetic rod is essentially an oscillator. When an alternating current is applied to the two poles of the actuator coil, the GMS rod will oscillate along its axial direction and generate mechanical energy. The conical energy-focusing head is designed to focus the mechanical energy generated by the supermagnetic rod.

To achieve higher energy transmission to the tested specimen, the cylindrical steel mass and the pre-tension screws are designed to apply appropriate prestress to the GMS rod. If the cylindrical steel mass and the pre-tension screws are removed, the back end of the GMS rod will be freed. When actuated by the driving alternating current, the GMS rod will extend and contract. According to the law of the conservation of momentum, the energy-focusing head and the GMS rod will move away from the tested specimen, and thus the energy cannot be transferred to the tested specimen. Conversely, if the cylindrical steel mass and the pre-tension screws are used, the GMS rod is equivalent to a fixed end. Ideally, the force between the energy-focusing head and the specimen is twice that of the stress generated by the GMS rod. However, the prestress will change the natural frequency of the whole structure and the excitation frequency. The efficiency of the energy transmission will be higher if the excitation frequency is the total structure’s natural frequency. Thus, the prestress level partly depends on the excitation frequency. The energy of the mechanical wave generated can be adjusted by changing the magnitude of the applied current.

**Figure 2:**
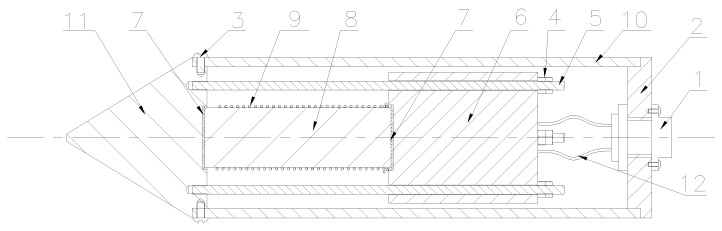
Structural diagram of the GMS ultrasonic actuator. (1) Plug seat; (2) Rear cover; (3) Screw; (4) Nut; (5) Pre-tension screws; (6) Cylindrical steel mass; (7) Backing gasket; (8) GMS rod; (9) Magnetic exciting coil; (10) Outer cover; (11) Energy-focusing head; (12) Wire to drive the circuit.

Figure 2 shows a detailed design schematic of the GMS ultrasonic actuator. To allow it to be held comfortably by a single hand, the diameter of the GMS actuator was chosen to be 50 mm. After the natural frequency of the GMS actuator and the material properties of components were confirmed, the dimensions of the other parts of the GMS actuator were calculated using the corresponding formulations as reported previously [59]. Table 1 lists the selected materials and the design size for each part of the GMS actuator. The GMS rod is a compound of rare earth elements, including a Tb–Dy–Fe alloy. This material is also named Terfenol-D, and was first developed in the 1970s. The extension rate of the GMS material is 10^−3^, which is four times better than that of piezoelectric materials. The energy density of the GMS material is $2\times 10^{4}\;J/m^{3}$, which is much higher than that of piezoceramic materials [60]. Furthermore, the GMS actuator has the advantages of a wide frequency range (100–10,000 Hz); stable, adjustable, and high energy generation; and good consistency in wave generation. The GMS actuator is superior to traditional guided wave excitation methods, such as hammer impact and piezoelectric-based methods. Hammer impact is inconsistent in the generation of energy and frequency for each excitation. The piezoceramic-based methods, which often require the use of a coupling agent, can only generate stress waves with small amounts of energy; thus the inspection range is very limited.

The design has several features: Firstly, the symmetrical design prevents irregularity of the excited stress waves. Secondly, the cylindrical steel mass amplifies the mechanical oscillations and ensures that the excited energy is propagated in the axial direction. Lastly and most importantly, the conical energy-focusing head increases the energy density [61], eliminating the requirement of using a coupling agent, which is needed when using a piezoceramic actuator.

### 3.2. Electrical Design

The designed GMS actuator is able to generate both coded pulse signals and sweep sine signals with the supporting electrical system, according to the inspection needs. The driving principles of these two signals are described below.

#### 3.2.1. Coded Pulse Signals

**Figure 3:**
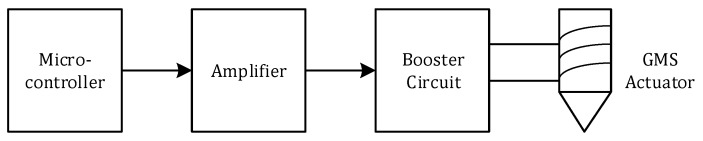
Principle of the coded pulse signal generator.

The pulse signal generator consists of a microcontroller STC12C5604AD (STC Micro Limited, Beijing, China), an amplifier, and a booster circuit, as shown in Figure 3.

As controlled through manual settings in the host computer, the microcontroller generates a variety of pulse signals, and the amplifier amplifies the pulse signal into a high-power current pulse. The booster circuit increases the voltage by 13 times, generating a 150 V pulse signal at a 12 V operating voltage, and a pulse of approximately 300 VPP at bipolarity. The GMS actuator then produces ultrasonic guided waves.

Therefore, the output of the coded pulse signals is determined by the logic level of P13 and P12. Five types of pulses can be generated; namely, (1) unipolar pulses; (2) bipolar pulses; (3) multipulses; and (4) coded combination pulses. The generation of the pulse can be single or periodic. The width of the pulse can be set between 2.5 μs and 1000 μs, the pulse interval can be set between 5 μs and 50 μs, and the number of pulses can be set between 1 and 250.

#### 3.2.2. Sweep Sine Signals

**Figure 4:**
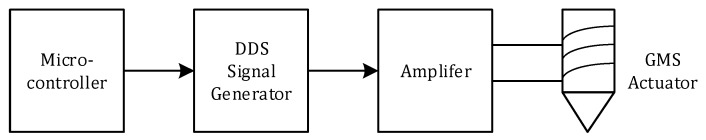
Principle of the sine sweep signal generator.

The sweep sine signal generator consists of a microcontroller (STC12C5604AD), a DDS signal generator (AD9851), and an amplifier, as shown in Figure 4.

Through manual settings in the host computer, the microcontroller controls the frequency range and sweep mode of the sweep sine signal, and transmits the signal to the DDS signal generator. The signal is then amplified before being fed to the GMS actuator. The core of the DDS signal generator is the AD9851 module (Analog Devices Inc., Norwood, MA, USA). The frequency range is 0.1 Hz–40 MHz. The initial frequency, step size of the scanning frequency, step interval, and the number of steps is adjustable. The generation of the signal can be single or periodic. The frequency range of the amplifier is 10 Hz–150 kHz. It has a maximum voltage output of 28 VPP, a maximum current output of 3 A, and a maximum power output of 20 W.

## 4. Experimental Verification

### 4.1. Experimental Setup

**Figure 5:**
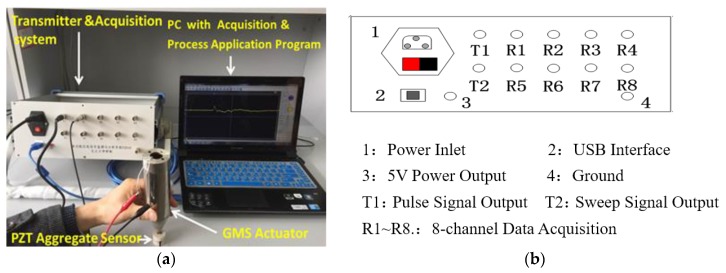
(**a**) Photo of the instrumentation and experimental setup; (**b**) The front panel of the transmission and acquisition system.

The testing system consists of a transmission and acquisition system, a computer with an acquisition and processing program installed, a PZT (lead zirconate titanate)-based smart aggregate as a sensor, and the GMS actuator. Figure 5a shows a photo of the instrumentation and experimental setup. The transmission and acquisition system is a self-developed piece of equipment, the front panel of which is shown in Figure 5b. The working principle of the transmitter module is as described in Section 3.2. The acquisition module is 16-bit and has a sampling rate of 2 MS/s. The sensor is a PZT-based smart aggregate encased by marble blocks, with a piezoelectric charge coefficient of 295.1 pC/N. In the setup, the computer controls the transmitter module via USB interface, and the transmitter module drives the GMS actuator via T1 or T2 port. The PZT-based sensor receives the guided waves, and the signals are acquired by the acquisition module and displayed on the computer. The PZT-based smart aggregate was manually attached to the exposed end of the rock bolt through a surface contact. Since the GMS actuator is able to generate stress waves with high power, no glue or coupling agent was needed for the PZT sensor to be able to detect the stress waves after many cycles of bouncing in the rock bolt.

### 4.2. Pulse Signal and Sweep Sine Signal Tests

**Figure 6:**
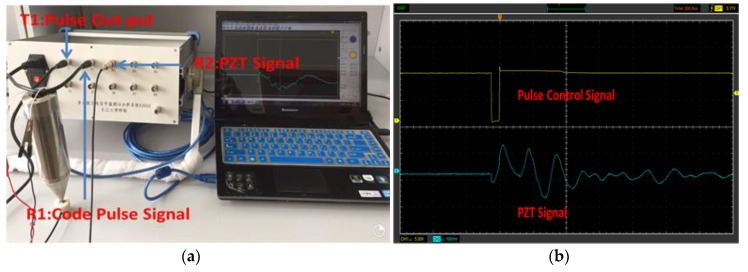
Pulse stress signal test. (**a**) Instrument setup for generating pulse signal; (**b**) Generated pulse signal waveform.

**Figure 7:**
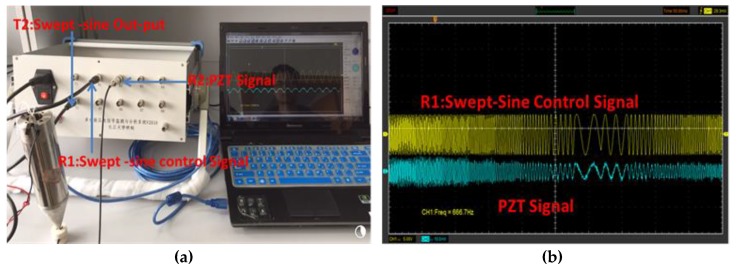
Sweep sine signal test. (**a**) Instrument setup for generating sweep signal; (**b**) Generated sweep signal waveform.

The setup for pulse signal transmission and reception is shown in Figure 6a, and the corresponding waveforms are shown in Figure 6b. The transmitted pulse is a 50 μs square pulse. The received wave shows several reflections of the pulse. Figure 7a shows the setup for the sweep sine signal transmission and reception, and the corresponding waveforms are shown in Figure 7b. The tested scanning signal has a frequency range of 300 Hz–40 kHz, considering the limitation of the amplifier (10 Hz–150 kHz). The correlation coefficient between the transmitted signal and the received signal is 88.7%; therefore, there is a good correlation between the transmitted signal and the received signal.

### 4.3. The Frequency Response of the GMS Actuator

**Figure 8:**
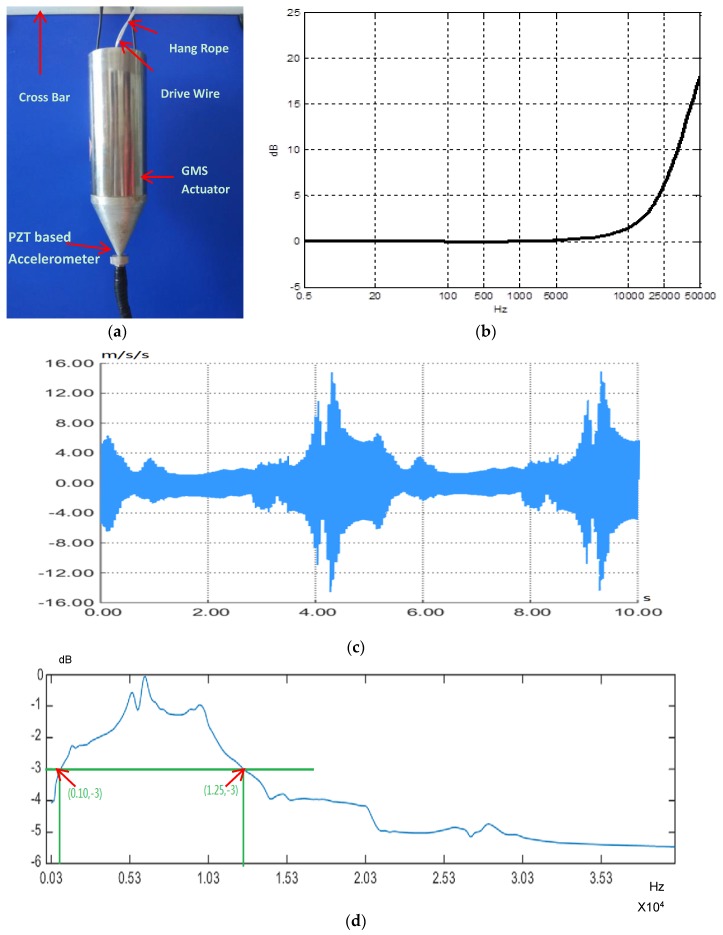
The frequency response spectrum testing for the GMS actuator. (**a**) The hanging GMS actuator; (**b**) The frequency response spectrum of the PZT sensor; (**c**) The sweep waveform; (**d**) The frequency response spectrum of the GMS actuator.

The experimental results shown in Figure 8b validate that the GMS actuator is able to generate a sweep signal. To obtain the frequency response of the GMS actuator accurately, a new experiment was performed. As shown in Figure 8a, the GMS actuator was hung in the air, and a PZT-based accelerometer with a better low-frequency response was used as a sensor, instead of the smart aggregate described in Section 4.1, to more accurately test the low-frequency characteristics of the GMS actuator. The PZT-based accelerometer integrates a traditional piezoelectric accelerometer with a charge amplifier to ensure better noise immunity. The PZT-based accelerometer has a sensitivity of 20.21 mV/(m·s^2^). The frequency response of the PZT-based accelerometer is shown in Figure 8b. The sweep signal was a 300 Hz–40 kHz signal with a duration of 5 s. Figure 8c shows the received sweep signal. Analyzing the power spectrum of the received signal and excluding the frequency response from the PZT sensor reveals the frequency response of the GMS actuator, as shown in Figure 8d. It is clear that the frequency range for –3dB is 1–12.5 kHz. The performance of the GMS actuator at low-frequency actuation is better than that of a PZT transducer [62], which makes it more suitable for large area inspections.

## 5. Application of the GMS Actuator to the NDE of Rock Bolts

### 5.1. Laboratory Case Study

#### 5.1.1. Specimen Fabrication

**Figure 9:**
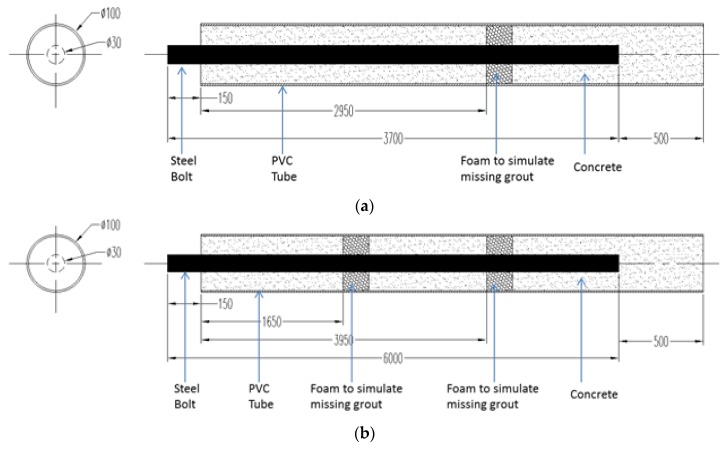
Illustrations of rock bolt specimens. (**a**) Specimen no. 1; (**b**) Specimen no. 2. PVC.

Rock bolts are the most important reinforcing elements in underground excavation, tunnel, and dam reinforcement. Failures of rock bolts occur due to overloading, corrosion, seismic activity, and poor grouting. We have fabricated two rock bolt specimens with missing grout. The rock bolt specimens consist of a steel bolt, a polyvinyl chloride (PVC) pipe, and concrete grout in the pipe, as shown in Figure 9. To simulate missing grout, a piece of foam was pre-embedded in a specific location before pouring concrete into the PVC pipe. Missing grout often results in rock bolt corrosion in the presence of moisture. The material properties and dimensions of these two specimens are listed in Table 2. To determine the wave velocity, a pulse signal was introduced to a 5 m steel rock bolt, and the reflected signal was recorded. The velocity was obtained by the dividing the travel length by the travel time.

#### 5.1.2. Instrumentation and Experimental Setup

**Figure 10:**
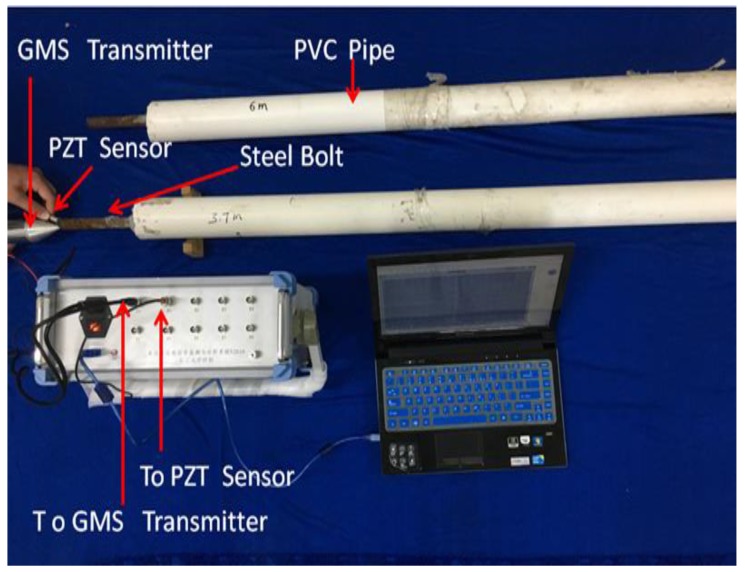
Laboratory setup for the NDE of rock bolts.

The instrumentation and experimental setup of the rock bolt testing are shown in Figure 10. The novel GMS actuator was used to generate ultrasonic guided waves to inspect the rock bolt, and the PZT sensor was used to receive the reflected guided waves. When testing rock bolt Specimen No. 1, a 40 V unipolar pulse with a width of 100 μs was used to excite the GMS actuator. To improve the signal energy and reduce attenuation, a 40 V unipolar pulse with a width of 500 μs was used when testing rock bolt Specimen No. 2. The sampling frequency was 1 MHz. Note that during the evaluation process, no coupling agent (such as ultrasonic gel) was needed, as a result of the special conic design of the actuator and the high-power actuation.

#### 5.1.3. Damage Estimation

As detailed in our previous work, the instant phase of the received waveform was obtained after removing noise and performing a Hilbert transformation [63]. The rock bolt length and defect information could then be obtained by analyzing the periodic characteristics of the instant phase. As expected, the wave energy generated by the GMS actuator was high, and was able to reflect at least three times along the rock bolt.

Assuming that the defect-induced instant phase change occurs at the $N_{th}$ sampling point, the defect location w can be expressed as

(1) $$w=\frac{N\Delta t\;v}{2}$$

where Δt is the sampling interval of the data acquisition system, and v is the velocity of the guided wave along the rock bolt direction. The defect location and length information is reflected in the sudden change in the instant phase. Supposing that the duration of the phase change is ΔT, the rock bolt length or defect location can be expressed as

(2) $$l=\frac{\Delta T\;v}{2}$$

**Figure 11:**
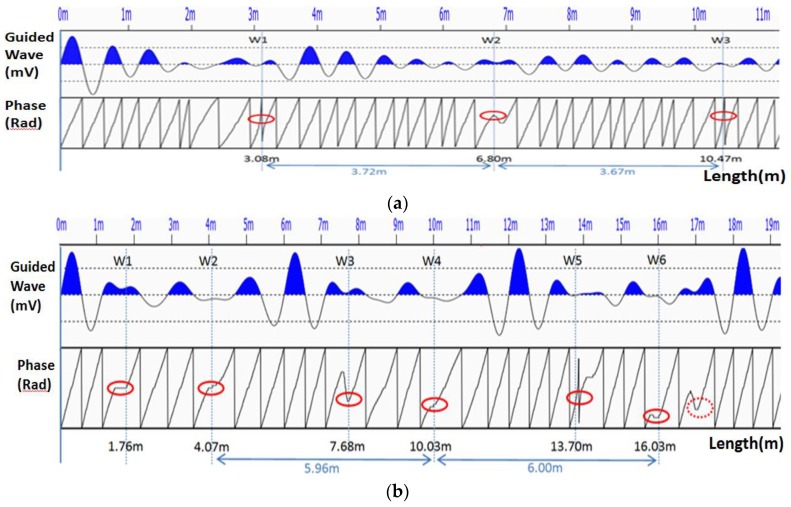
The received waveform and its instant phase. (**a**) Specimen No. 1; (**b**) Specimen No. 2.

Once the full length of the rock bolt is obtained, the defects can be identified as the locations that are at points shorter than the full length. As an example, the received waveform and the instant phase of specimen no. 1 are shown in Figure 11a. The sudden change in the instant phase is marked by the red circle. According to Equation (4), the locations of the instant phase change are: $w_{1}=3.08\;m$, $w_{2}=6.80\;m$, $w_{3}=10.47\;m$. Since $w_{2}-w_{1}\approx w_{3}-w_{2}$ ($w_{2}-w_{1}=3.72\;m,\;w_{3}-w_{2}=3.67\;m$), the change of the instant phase is periodic. Therefore, the length of the rock bolt is

(3) $$l=\left((w_{2}-w_{1})+(w_{3}-w_{2}\right))/2=3.70\;m$$

Since $w_{1}<l$, it can be determined that $w_{1}=3.08\;m$ is the defect location. The detected defect location is in good agreement with the experimental setup.

The received waveform and the instant phase of Specimen No. 2 are shown in Figure 11b. There are two instant phase-changes within one period, which agrees with the experimental setup. The rock bolt length can be obtained by

(4) $$l=\left((w_{4}-w_{2})+(w_{6}-w_{4}\right))/2=6.00\;m$$

The locations of $w_{1}$ and $w_{2}$ indicate the two defect locations, which are at 1.76 m and 4.07 m, respectively. The results agree well with the experimental setup.

### 5.2. Field Implementation

**Figure 12:**
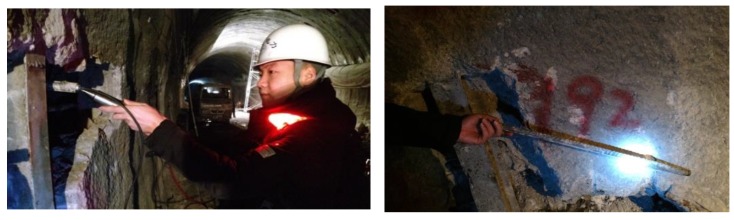
Field testing and verification by retrieval.

The proposed GMS actuator was successfully demonstrated in several field-implementation projects. During 20–22 December 2016, the GMS system was implemented in the inspection of rock bolts in a hydroelectric station located in Zhouqu County, Gansu Province, China. The goal was to estimate the length of the installed rock bolts. Figure 12 shows that the field testing was conducted by using the developed GMS actuator, and then retrieving the rock bolt to verify its length. In total, 206 rock bolts were tested; 45 of which were randomly chosen and retrieved to verify their actual lengths. Table 3 lists the measured lengths and actual lengths of 10 of the rock bolts. Evidently, the error is low. The average relative error is 2.6%, and the absolute error is less than 2 cm. The error can be further reduced by increasing the sampling rate of the system.

**Figure 13:**
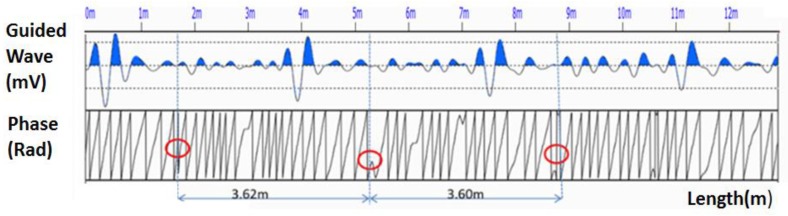
The length extraction process of rock bolt No. 203.

The measurement of rock bolt No. 203 was taken as an example. Figure 13 shows the waveform and the length extraction process. According to the change in the instant phase and the calibrated wave propagation velocity, the length of rock bolt No. 203 was (3.62 + 3.60)/2 = 3.61 m. The actual length of the bolt from retrieval verification is 3.59 m. The absolute error is 2 cm and the relative error is 0.6%.

It is apparent that there are also phase jumps at 3 m, 6 m, and 7 m. There are several factors, such as random disturbance and ambient noise, that can cause these phase jumps. However, the phase jumps caused by these factors will not show the periodic characteristics that are observed for the jumps caused by defects and the end reflection. Therefore, there are two necessities for the identification of the length and the defect location: (1) the phase change must be sudden; and (2) the phase change must be periodic. The phase jumps at 3 m, 6 m, and 7 m do not show any periodic characteristics, and thus cannot be considered as the location of the defects nor of the end reflections. If there are defects in the rock bolt system, there will be two subsequent phase jumps induced by the defect and the end reflection; both of them will have the same period, and the one that shows an earlier arrival is the one induced by the defect.

### 5.3. Discussion

Results from the laboratory verification and field implementation show that the proposed GMS actuator and system is able to detect rock bolt damage and measure the installed rock bolt length accurately, in a nondestructive way. The proposed GMS-based guided wave system differentiates itself from many other systems in these aspects: (1) The system offers high actuation energy, thus the induced stress wave can travel many cycles in the evaluation specimens, in order to obtain accurate evaluation results. (2) The system is portable and easy to use, and there is no need to use a coupling agent. (3) The system has a wide frequency band, and the excitation signal is adjustable. For small-sized objects, a high excitation frequency can be used; and for large size objects, a low excitation frequency can be adopted. (4) The output power of the system can be adjusted. For small-sized objects, low energy excitation can be used to avoid signal saturation and distortion; and for large-sized objects, high energy excitation can be adopted to ensure high signal-to-noise ratio. (5) The consistency of the repeated measurement is high; thus, errors due to operational error and environmental noise can be greatly reduced. (6) As demonstrated in the field tests, the system is robust and can be operated in harsh environments, such as in a tunnel during construction work.

In addition, with proper modification, the proposed guided wave actuator may also be applied to many other applications, such as the quality assessment of bridge anchor cables, foundation piles, and other interface issues related to concrete and steel structures; geological surveying; and the acoustic characteristic of wells.

## 6. Conclusions

The excitation method of ultrasonic guided waves plays an important role in the advancement of guided wave testing technology. Based on the giant magnetostrictive (GMS) effect, a novel guided wave excitation device and system were developed in this paper. The innovative GMS device has the advantages of large force generation, adjustable output power, wide bandwidth, low requirement on coupling, and simple operation. The developed GMS actuator is superior to traditional guided wave excitation methods, such as hammer impact and piezoelectric-based methods. The GMS device is implemented by using pulse code modulation (PCM) and direct digital synthesis (DDS) driving technology. The device supports two kinds of output modes: pulse coding and sweep signals. The device was applied in the missing grout defect assessment of rock bolts. The results showed the effectiveness and reliability of the proposed method. The proposed actuator and system may also be used in other guided wave-based inspection applications.

## Figures and Tables

**Table 1:** Materials in the components of the GMS actuator.

Component Name	Material	Component Size	
		Diameter	Length
GMS rod	GMS (Terfenol-D)	10 mm	70 mm
Cylindrical steel mass	stainless steel	40 mm	40 mm
Magnetic exciting coil	silver, copper	20 mm	70 mm
Energy-focusing head	aluminum	50 mm	40 mm
Outer cover	stainless steel	50 mm	140 mm

**Table 2:** Parameters of the specimens.

Specimen No.	Wave Velocity in Steel Bolt (m/s)	Steel Bolt Diameter (mm)	Steel Bolt Length (mm)	PVC Pipe Diameter (mm)	Concrete Class	Defect Location (mm)
1	5100	30	3700	100	C30	3100
2	5100	30	6000	100	C30	1800 and 4100

**Table 3:** Comparison of measured and actual lengths.

No.	2	47	80	88	91	94	102	111	192	203
Measured length (m)	2.39	0.12	1.74	0.33	2.30	0.25	2.56	3.23	1.00	3.61
Actual length (m)	2.40	0.13	1.75	0.35	2.32	0.27	2.55	3.25	1.02	3.59
Absolute error (cm)	1	1	1	2	2	2	1	2	2	2
Relative error (%)	0.4	7.7	0.6	5.7	0.9	7.4	0.4	0.6	2	0.6
Average error (%)	2.6%									
